# Balancing the Photoreceptor Proteome: Proteostasis Network Therapeutics for Inherited Retinal Disease

**DOI:** 10.3390/genes10080557

**Published:** 2019-07-24

**Authors:** Siebren Faber, Ronald Roepman

**Affiliations:** Department of Human Genetics and Radboud Institute for Molecular Life Sciences, Radboud University Medical Center, Geert Grooteplein Zuid 10, 6525 GA Nijmegen, The Netherlands

**Keywords:** protein trafficking, protein folding, protein degradation, chaperones, chaperonins, heat shock response, unfolded protein response, autophagy, therapy

## Abstract

The light sensing outer segments of photoreceptors (PRs) are renewed every ten days due to their high photoactivity, especially of the cones during daytime vision. This demands a tremendous amount of energy, as well as a high turnover of their main biosynthetic compounds, membranes, and proteins. Therefore, a refined proteostasis network (PN), regulating the protein balance, is crucial for PR viability. In many inherited retinal diseases (IRDs) this balance is disrupted leading to protein accumulation in the inner segment and eventually the death of PRs. Various studies have been focusing on therapeutically targeting the different branches of the PR PN to restore the protein balance and ultimately to treat inherited blindness. This review first describes the different branches of the PN in detail. Subsequently, insights are provided on how therapeutic compounds directed against the different PN branches might slow down or even arrest the appalling, progressive blinding conditions. These insights are supported by findings of PN modulators in other research disciplines.

## 1. Introduction

The rod and cone photoreceptor (PR) cells are the most abundant cell types in the human retina, with ~6.4 million cones and up to 125 million rods per adult retina [1]. PRs are highly specialized, polarized neurons of neuroepithelial origin, consisting of morphologically and functionally distinct cellular compartments, including a synaptic terminal, an inner segment (IS), an outer segment (OS), and a connecting cilium bridging the IS and OS. The classical division into rods and cones is based on their different OS morphology and differential expression of subtypes of opsin. The rod outer segments are long, thin, rod-shaped organelles containing rhodopsin in large stacks of membranous discs, allowing to process variations in dim-light conditions, but they lose this ability in bright light conditions. The OSs of cones represent a shorter conical organelle containing either S-opsin, M-opsin, or L-opsin, which are less sensitive compared to rhodopsin, but are perfectly suited to process bright light of different wavelengths, allowing color vision [2].

Despite the difference in photosensitivity, the process of converting light stimuli into biochemical signals, the phototransduction cascade, is almost indistinguishable between rods and cones [3,4]. In rods, upon capture of a photon, the chromophore 11-*cis* retinal, which is conjugated to a rhodopsin molecule, undergoes a conformational change that isomerizes it to all-*trans* retinal leading to the activation of rod opsin. The activated opsin molecule is now able to bind and subsequently activate the G-protein, transducin. Transducin consists of three subunits: G_α_, G_β_, and G_γ_. In its inactive state, G_α_ is bound to a GDP. When transducin gets activated by rhodopsin, this GDP is replaced by a GTP and subsequently G_α_ dissociates from the G_βγ_ subunit. The GTP bound G_α_ is now able to displace one of the two inhibitory γ-subunits of cGMP-specific 3′,5′-cyclic phosphodiesterase (PDE6) causing the exposure of one of the two catalytic sites of the PDE6_αβ_ heterodimer (PDE6_α’_ for cones). The exposed catalytic sites are now able to hydrolyze multiple cGMP molecules to 5′-GMP. In the dark state, cGMP binds to its specific cation channel allowing a steady movement of cations into the OS, which is compensated by the efflux of cations from the OS by Na^+^/Ca^2+^, K^+^ exchangers (NCKXs) [5]. Due to the hydrolysis of cGMP, its levels decrease causing the closure of cation channels in the OS. The resulting hyperpolarization of the OS membrane spreads throughout the cell, eventually reaching the synaptic terminal. Here, the hyperpolarization causes closure of calcium channels and subsequently a decrease in calcium-dependent synaptic glutamate release [3,4], which activates the glutamate receptors of the bipolar cells postsynaptically, transducing the brain-bound electrical signal [6].

The phototransduction cascade, carried out in only a few milliseconds, is based on capture of one single photon in dim light. However, in bright daylight conditions, immense amounts of photons are caught by the human PRs, mainly cones, every second, which causes toxic photo-oxidative damage to the OS components. These components, which include most proteins involved in the phototransduction cascade, are therefore continuously renewed (~10% every day for human PRs), by incorporating newly synthesized PR discs at the OS base, and shedding the oldest membrane discs at the cell’s tip [7,8]. The shed OS tips are subsequently phagocytosed by the retinal pigment epithelium (RPE) cells, which is the highest active phagocytic process in our body [9,10]. Because of this highly active protein turnover, a mutation in a gene encoding a protein involved in this machinery most often leads to PR cell death, a classical hallmark of many inherited retinal diseases (IRDs).

IRDs are a genetically heterogeneous group of rare eye disorders with a progressive manifestation causing vision loss or even complete blindness [11]. Mutations in many genes that are required for PR function were found to be causative for IRDs. These include genes encoding proteins involved in protein trafficking, splicing, energy metabolism, and photoreceptor structure [12]. The compartments of PRs most prominently implicated in IRDs are the OS and the connecting cilium. The connecting cilium correlates with the transition zone of a primary cilium and functions as a gatekeeper to regulate the molecular composition of the OS. Thus, the OS, connecting cilium, and the immediately adjacent basal body, form the photoreceptor sensory cilium, one of the most highly specialized primary cilia, fully optimized for photoreception and transduction [13,14]. Hence, IRDs with defects in these compartments are referred to as retinal ciliopathies [11].

Defects in the conserved ciliary processes mostly manifest as syndromic ciliopathy conditions, affecting multiple organs. The most prominent ciliopathies in which the retina is affected are Bardet–Biedl syndrome (BBS), Joubert syndrome (JS), Meckel–Gruber syndrome (MKS), and Senior–Løken syndrome (SLS) [4,11]. Because PRs are highly specialized, polarized neurons, retinal ciliopathies often also occur in a nonsyndromic fashion, affecting ciliary proteins specific to rods and/or cones. Based on the PR cell type affected, different IRDs can be categorized, including retinitis pigmentosa (RP), Leber congenital amaurosis (LCA), cone-rod dystrophy (CRD), and achromatopsia (ACHM) [4,11].

RP is the most common form of inherited blindness worldwide, with a prevalence of about 1:4000 people. The cells that are affected initially in RP are the rods leading to night blindness and loss of peripheral vision. Secondary, cones are most often also affected as the disease progresses [15].

LCA is the most severe form of inherited retinal degeneration, causing patients to suffer from severe visual impairment to complete blindness before the first year of life. With LCA, both rods and cones are affected at early stages often leading to complete blindness [16].

Another early onset form of hereditary retinal dystrophy is CRD, which manifest in the opposite order compared to RP, since in CRD cones are initially affected, followed by the loss of rods [17].

A disease in which specifically the cones deteriorate is ACHM, most often leading to complete color blindness [18].

Apart from some specific exceptions, most of the above described blinding conditions are still considered incurable. However, significant and promising progress has been made with therapeutic studies targeting specific genes and/or mutations, most prominently by employing gene augmentation and antisense-oligo nucleotide (AON)-based techniques [19,20]. Yet, a major disadvantage of these treatments is that they are complex and invasive for the patient. On top of that, for nearly every mutation, a personalized approach needs to be developed. Therefore, there is a high interest in pharmacological agents with a broad range of disease targets. To discover such agents, a growing number of studies have focused on a common phenomenon seen in many IRDs before the actual death of rods and cones: the accumulation and/or misfolding of proteins in the inner segment of the photoreceptors [21].

**Figure 1:**
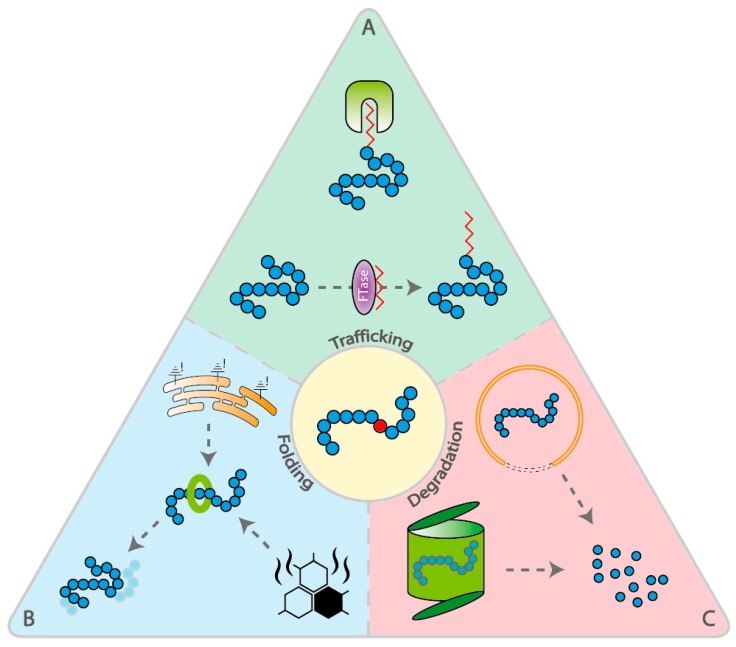
Schematic representation of the different branches of the proteostasis network, divided into trafficking, folding and degradation. (A) Trafficking: Protein prenylation involves the transfer of either a farnesyl or a geranylgeranyl moiety to the C-terminal cysteine(s) of the target protein by farnesyltransferase (FTase) and geranylgeranyltransferase (GGTase-I), respectively. Chaperones involved in regulation of this prenylation process and trafficking of prenylated proteins, include AIPL1, PDE6D, and UNC119 (depicted in green). (B) Folding: Two responses that are activated upon protein misfolding are the unfolded protein response (UPR) and the heat shock response (HSR). Both responses result in enhanced expression of chaperones (depicted in green) in order to restore the correct folding of proteins. (C) Degradation: ER-associated degradation (ERAD) results in the degradation of proteins by the ubiquitin–proteasome system (UPS). Another pathway of protein degradation is autophagy. Background colors in figure (green, blue, and red) correspond with background colors of Table 1.

This phenomenon can be explained by the malfunction of several processes, all involved in protein homeostasis or proteostasis. A first process is the disruption of protein transport from the IS to the OS (Figure 1A). As the OS lacks the biosynthesis machinery for proteins and lipids, all of the components required for OS morphogenesis, maintenance, and sensory functions must be transported from the endoplasmic reticulum (ER), located in the IS, to the OS [2], across the connective connecting cilium stalk. Therefore, defects in this transport most often lead to the accumulation of proteins in the IS. Another process involved in the accumulation of proteins in the IS is the dysregulation of pathways involved in protein degradation, including ER-associated degradation (ERAD) and autophagy (Figure 1C). Finally, also incorrect folding of specific proteins due to loss-of-function mutations can results in protein accumulation in the IS (Figure 1B). An accumulation of misfolded proteins in the ER membrane or in the cytoplasm of the PRs can induce the heat shock response (HSR) and/or the unfolded protein response (UPR). Continuous activation of these responses will ultimately lead to death of PRs [21].

In this review the above mentioned branches of the proteostasis network will be described in detail. Subsequently, we will explain how potential therapeutic agents directed against these branches might slow down or even arrest the dramatic blinding conditions, supported by findings of PN modulators in other research disciplines.

## 2. The Different Branches of the Proteostasis Network

### 2.1. Pathways Involved in Protein Trafficking

#### 2.1.1. Chaperones Involved in Lipid-Dependent Trafficking

Many PR-specific proteins depend on post-translational lipid modifications, including prenylation and myristoylation, for correct localization to the OS and their anchoring at one of the OS membranes [22]. Protein prenylation involves the transfer of either a farnesyl or a geranylgeranyl moiety to the C-terminal cysteine(s) of the target protein by farnesyltransferase (FTase) and geranylgeranyltransferase (GGTase-I), respectively [23]. Several chaperones are involved in this prenylation process, including AIPL1, PDE6D, and UNC119.

Aryl hydrocarbon receptor (AhR)-interacting protein-like 1 (AIPL1) was first discovered in association with Leber congenital amaurosis (LCA) [24]. Mutations in the *AIPL1* gene encoding for AIPL1 results in LCA4, one of the most severe forms of LCA leading to blindness in early childhood [25]. Since then, studies were set up to unravel the function of the AIPL1 protein. Studies in AIPL1 knockdown and knockout mouse models revealed that AIPL1 functions as a chaperone for PDE6 [26,27]. In these mouse models protein levels and activity of PDE6 were severely reduced, whereas other photoreceptor proteins were unaffected. Other studies suggest that AIPL1 is also necessary for the maintenance of PRs by enhancing the essential farnesylation reaction [28,29]. Several retinal proteins, including PDE, transducin, and rhodopsin kinase (GRK1) are known to be farnesylated [30]. These findings are in line with the observed rapid degeneration of rods and cones in the animal models, as well as in LCA4 patients. 

Another chaperone, mainly involved in the trafficking of prenylated proteins, is PDE6D [8,31]. In mice, knockout of PDE6D results in mislocalization of prenylated PR proteins, including PDE6, GRK1, and Gγ [32]. In human, mutations in PDE6D are associated with Joubert syndrome (JS) [33,34]. This syndromic condition can be explained by mislocalization of other critical cargos of PDE6D important for the development and stability of the primary cilium. These cargos include inositol polyphosphate 5-phosphatase E (INPP5E) and retinitis pigmentosa GTPase regulator (RPGR) [33,35]. The trafficking of these cargos comprises of several steps [36,37]. First, PDE6D solubilizes the prenylated protein from the ER by sequestering the hydrophobic farnesyl or geranylgeranyl tail. Subsequently, PDE6D targets the cargo to the destination membrane by association with a peripherally membrane bound docking protein. Finally, binding of the displacement factor ARL3 to PDE6D causes a reduction in size of the hydrophobic cavity resulting in the release of the cargo [38]. PDE6D can now travel back to repeat its cycle.

UNC119 shares significant sequence and structural homology with the prenyl-binding protein PDE6D [37]. Similar to PDE6D, UNC119 is also a subject of ARL3-dependent cargo release, and therefore the transport mechanism of PDE6D and UNC119 are highly identical. However, a remarkable difference between PDE6D and UNC119 is the ARL3-dependent release of their cargo. In contrast to PDE6D, the hydrophobic pocket of UNC119 is expanded upon binding of ARL3, thereby weakening the interaction with the cargo instead of squeezing the lipid out of the hydrophobic pocket [39]. This might be partly explained by the binding of different lipid moieties of both chaperones. Whereas PDE6D sequesters prenylated proteins, UNC119 is known to bind myristoylated cargo [31,40]. UNC119 has been shown to be important in the trafficking of transducin, which is myristoylated on its α-subunit [41]. In UNC119 knockout mice, the trafficking of transducin to the OS during dark adaptation was significantly impaired, contributing to the observed slow retinal degeneration [42]. The observed mislocalization of transducin in these mice is not complete, supporting the fact that transducin is trafficked by other proteins, including PDE6D. Interestingly, a study performed in *Pde6d;Unc119* double-knockout mice showed partially improved rhodopsin kinase (GRK1) expression and trafficking in cones compared to *Pde6d* single-knockout mice [43]. Based on these findings, the authors suggest that the transport by PDE6D and UNC119 in cones might be interdependent. The improvement found in only cones is in line with the relatively mild phenotype seen in patients with a truncating mutation in UNC119, which is associated with late onset CRD [44].

### 2.2. Pathways Involved in Protein Folding

#### 2.2.1. Chaperonins, Phosducins and Ric8A

In addition to the classic and ubiquitous molecular chaperones from the heat shock protein superfamily (discussed below), various studies have focused on chaperones and co-chaperones involved in folding and assembly of PR-specific proteins. 

One of the PR-specific proteins that needs targeted guidance for its correct folding and assembly is transducin. The folding and assembly of the full transducin protein complex is performed in two distinct steps. In the first step, the so-called chaperonins or CCTs (chaperonins containing TCP-1) are involved. CCTs are protein-folding ATPase complexes consisting of two stacked rings, which form a central cavity [45]. The β-subunit of transducin (G_β_) is able to enter this cavity. Once transducin enters, ATP binding occurs, resulting in the closure of the cavity by hydrolysis of the just bound ATP molecule [46]. In addition to ATP, the folding of G_β_ requires a co-chaperone, phosducin-like protein 1 (PhLP1). Binding of PhLP1 induces conformational changes of CCT that lock the β-propeller structure of G_β_. Subsequently, G_β_ rotates inside the cavity followed by its release in complex with PhLP1. PhLP1 is required for the formation of the G_βγ_ dimer [47]. 

The second step involves the trafficking of the G_βγ_ dimer to the OS by phosducin, another member of the phosducin family of proteins. Phosducin forms a complex with G_βγ_ by sequestering the hydrophobic farnesyl residue of G_γ_ [48]. The farnesyl residue serves as a membrane anchor, which targets G_βγ_ to the OS [49]. Another speculated function of phosducin is the protection of G_βγ_ against ubiquitination and degradation by the proteasome [50]. 

CCTs are also involved in the folding and assembly of the BBSome [51], a protein complex that regulates the ciliary import, export, and intraciliary trafficking of molecules [52]. Mutations in genes coding for BBSome proteins most often result in the development of Bardet–Biedl syndrome, one of the syndromic ciliopathy conditions in which the retina is affected [53], as indicated in the introduction. For some of the BBSome proteins it was shown that these proteins contain a β-propeller fold, similar to G_β_ [54]. Whether or not the folding and assembly of the BBSome proteins is comparable to the situation of G_β_ remains to be elucidated.

It remains largely unknown if the α-subunit of transducin also needs assistance in correct folding and trafficking to the OS before it forms a heterotrimeric complex with G_βγ_. However, evidence is emerging that resistance to inhibitors of cholinesterase 8 (RIC8) proteins function as ubiquitous chaperones for G_α_ [55]. The two known isoforms—RIC8A and RIC8B—each regulate specific classes of G_α_ [56]. Although the specific PR class of Gα and its relation to RIC8 has not been investigated, it has been shown that a closely related form interacts with RIC8A [57]. The proposed mode of action of RIC8A is to positively regulate G_α_ by acting as a guanine nucleotide exchange factor (GEF). RIC8A preferentially binds GDP-bound G_α_ and causes GDP release. Subsequently, RIC8A assists in organizing a de novo nucleotide-binding pocket for binding of GTP. The RIC8A-G_α_ complex breaks apart when a GTP molecule binds. Subsequent hydrolysis of GTP results in the binding of G_α_ to the G_βγ_ yielding the fully assembled heterotrimeric complex [58]. Taken together, CCT together with PhLP1 regulate the folding of the transducin β-subunit. Subsequently, G_β_ forms a dimer with G_γ_ by guidance of PhLP1. Finally, the G_βγ_ dimer is trafficked to the OS by phosducin, where it can bind to G_α_. RIC8A has been proposed as the chaperone of G_α_ and its assembly to the G_βγ_ dimer.

#### 2.2.2. Heat Shock Response (HSR)

The HSR is known to be present in every living organism, and functions as an essential survival mechanism against extracellular challenges, such as increased temperatures, and intracellular stressors such as oxidative stress, that lead to protein misfolding [21,59]. The HSR is featured by an extremely rapid activation of gene expression, leading to a remarkable increase in molecular chaperones, including heat shock proteins (HSPs) [60]. In vertebrates, the HSR is regulated by a family of transcription factors, which includes six members (HSF1-4, HSFX, and HSFY) [59]. HSF1 is believed to be the master regulator of molecular chaperone synthesis upon protein misfolding [61]. Under normal physiological conditions, monomeric HSF1 is repressed and localized in the cytosol by interacting with molecular chaperones, including HSP90, HSP70, and the chaperonin CCT. When a threshold of misfolded proteins is reached, the chaperones dissociate from HSF1 leading to trimerization and nuclear accumulation of HSF1 followed by transcriptional activation of HSPs. The major HSPs involved in the HSR include HSP90, HSP70, and HSP60 [61].

HSP90 and HSP70 are able to associate with the hydrophobic regions of the protein, and thereby preventing binding to other hydrophobic moieties [61]. Subsequently, they assist in the folding of these hydrophobic regions in such a way that the hydrophobic groups are not exposed. Furthermore, they aid in overcoming the energy thresholds of the intermediate folding states of the protein [62]. Similar to HSP90 and HSP70, the chaperonin HSP60 is also able to bind hydrophobic residues of proteins. In contrast, the barrel-shaped HSP60 is more passively involved in the folding of proteins by creating a favorable microenvironment for the hydrophobic regions in its central cavity [63].

#### 2.2.3. Unfolded Protein Response (UPR)

A more specific cellular stress response related to the ER is the UPR. The UPR is activated in response to an accumulation of unfolded and/or misfolded proteins in the lumen of the ER [64]. The activation of the UPR aims to reduce ER stress and restore proteostasis in three distinct ways: an initial response to reduce protein synthesis, a second wave of increased production of molecular chaperones involved in protein folding to increase folding capacity, and finally the degradation of misfolded proteins when the folding capacity is insufficient to restore functional protein balance. It is generally accepted that a member of the HSP70 family, BiP/GRP78, is the key regulator in the activation of the UPR [65]. BiP has been shown to associate with all three transmembrane sensors of the UPR, including inositol requiring enzyme-1 (IRE1) α and β, protein kinase RNA (PKR)-like ER kinase (PERK), and activating transcription factor 6 (ATF6) α and β. Under normal physiological conditions, BiP associates with the ER luminal domain of IRE1 and PERK via its ATPase domain, maintaining them in an inactive monomeric state. 

The interaction of BiP with ATF6 masks a binding site to coat protein-II (COP-II) vesicles, thereby preventing ATF6 translocation to the Golgi. Upon ER stress, BiP dissociates from the sensors and binds to misfolded proteins. Subsequently, IRE1 and PERK are able to oligomerize followed by their trans-autophosphorylation and ATF6 traffics to the Golgi for further processing and activation. 

Dissociation of BiP from IRE1 results in the activation of its RNase domain. This activation is sustained by binding of HSP47 to the luminal domain of IRE1, thereby hindering the binding of BiP [66]. Subsequently, a single mRNA encoding for X-box binding protein 1 (XBP1) is targeted for non-conventional splicing [67]. 

These spliced XBP1s trigger the expression of molecular chaperones and components of ERAD in order to relieve the load on the ER and eventually restore protein balance [68]. One of the upregulated chaperones is the ER resident co-chaperone ERdj4, which eases and stabilizes the binding of BiP [69]. Furthermore, it has been shown that protein disulfide isomerase A6 (PDIA6) plays an important role in the conversion of IRE1 to its monomeric state [70]. Based on these findings, it is speculated that ERdj4 and PDIA6 are important regulators of the IRE1 negative feedback loop. Activation of PERK leads to phosphorylation of the α-subunit of eukaryotic translation initiation factor 2 (eIF2α), thereby hindering the conversion of eIF2-GDP to eIF2-GTP by the GEF eIF2B [71]. The inactivation of eIF2α leads to overall decrease in mRNA translation and thus protein synthesis. Although, global mRNA translation is reduced, some specific mRNAs are favored to be translated, including activation transcription factor 4 (ATF4) [72]. ATF4 is able to activate the transcription of various genes involved in protein folding, autophagy, and apoptosis, including CCAAT/enhancer-binding protein (C/EBP) homologous protein (*CHOP*), ER oxidoreductin 1 (*ERO1*), and growth arrest and DNA damage-inducible protein (*GADD34*). GADD34 plays a role in the negative feedback loop for PERK by acting as a cofactor in the dephosphorylation of eIF2α [73]. CHOP and ERO1 mainly play a role in prolonged PERK activation [74], which will be discussed below.

Under ER stress, ATF6 binds to COP-II vesicles and traffics to the Golgi [75]. Here, it is cleaved by proteases S1P and S2P resulting in a cytosolic fragment [76]. This fragment travels into the nucleus to regulate transcription of specific UPR genes, including *CHOP* and *XBP1*. Interestingly, XBP1s and ATF6 are able to heterodimerize, and subsequently induce the expression of genes involved in ERAD [77].

### 2.3. Pathways Involved in Protein Degradation

#### 2.3.1. ER-associated Degradation (ERAD)

Folding and maturation of proteins is a complicated process that is highly susceptible to errors. Up to 30% of all newly synthesized proteins are affected by such errors and are therefore degraded by the proteasome before they reach their defective mature state [78]. An important player in proteasomal-dependent protein degradation is ERAD. The process of ERAD can be divided into three consecutive steps: recognition of misfolded proteins in the ER, retrotranslocation of these proteins into the cytosol, and ubiquitin-dependent degradation by the ubiquitin–proteasome system (UPS) [79].

The recognition of misfolded proteins depends on the detection of substructures within proteins, including exposed hydrophobic regions, unpaired cysteine residues, and immature glycans [80]. The latter involves the lectin-type chaperones calnexin and calreticulin, which can bind to glycans possessing the Glc_3_Man_9_GlcNAc_2_ structure produced by deglucosylation [81]. Incompletely or misfolded proteins undergo multiple rounds of reglucosylation by uridine diphosphate (UDP)-glucose:glycoprotein glucosyltransferase (UGGT) and subsequent folding by reassociation with calnexin or calreticulin, eventually resulting in the mature protein conformation [82]. 

On the other hand, terminally misfolded proteins must be extracted from this calnexin/calreticulin cycle. This extraction is regulated by the removal of terminal mannose residues from core glycans by mannosidases, including ER mannosidase I (ERMANI), ER degradation-enhancing α-mannosidase-like protein 1 (EDEM1), EDEM3, or Golgi-resident mannosidase α class 1C member 1 (MAN1C1) [83,84,85]. In this way, ERAD can discriminate between terminally misfolded proteins and their fully functional counterparts. 

Since the ubiquitin-dependent degradation by the UPS is performed in the cytosol, terminally misfolded proteins subjected to ERAD have to be transported through the ER membrane via a transmembrane channel, called the retrotranslocon [86]. The exact composition of this channel is yet unknown. However, it has been shown that the membrane protein Derlin-1 and various E3 ubiquitin ligases are part of the retrotranslocon complex [87]. Once a small part of the substrate for ERAD is exposed to the surface of the ER, the substrate becomes a subject for poly-ubiquitination. Subsequently, the p97/valosin-containing protein (VCP), which is a member of the type II AAA+ protein family of ATPases, together with its cofactors, possessing ubiquitin-binding domains (UBDs), cooperatively produce a driving force for the retrotranslocation [88]. 

Retrotranslocated substrates for ERAD often possess exposed hydrophobic domains. Therefore, it is important that these substrates should be rapidly transported to the UPS for degradation to prevent aggregation. A chaperone complex consisting of BCL-2-associated athanogene 6 (BAG6), ubiquitin-like protein 4A (UBL4A), transmembrane domain recognition complex 35 (TRC35), and co-chaperone small glutamine-rich TPR-containing protein α (SGTA) has been shown to play an important role in the trafficking of ERAD substrates to the UPS by binding to the hydrophobic domains [89].

#### 2.3.2. Autophagy

Another very important process involved in protein degradation is autophagy. Multiple forms of autophagy are known, including macroautophagy, chaperone-mediated autophagy (CMA), and microautophagy [90].

Macroautophagy is the most common form of autophagy and therefore often referred to simply as autophagy. Autophagy consists of multiple consecutive phases, including initiation, nucleation, elongation, closure, and fusion to the lysosome. The initiation phase is tightly regulated by AMP-activated protein kinase (AMPK) and mammalian target of rapamycin complex I (mTORC1) [91]. In addition, factors expressed resulting from ER stress are also able to trigger the initiation phase. 

It all starts with the formation of the Unc-51-like kinase 1 (ULK1) complex followed by the phosphorylation of the class III phosphoinositide 3-kinase (PI3KC3) complex I leading to the nucleation of the phagophore [91,92]. Subsequently, autophagosome elongation and closure requires two ubiquitin-like conjugation systems, including an ATG7 dependent system and an ATG5-ATG12 dependent system. Together they are responsible for the lipidation, by phosphatidyethanolamine (PE), from cleavage of microtubule-associated protein 1 light chain 3 (LC3). Membrane sources in form of lipids needed for the elongation of the autophagosomal membrane are provided by ATG9-dependent vesicle trafficking. After closure of the autophagosomal membrane, the autophagosome has to fuse with the lysosome to form an autolysosome in order to degrade its enclosed cargo [93].

A selective form of autophagy is CMA, which to date is only identified in mammals [94]. This form of autophagy is specific for proteins containing a KFERQ motif in their amino acid sequence [95]. Heat shock cognate of the HSP70 family, HSC70, is able to recognize this motif and subsequently targets these proteins to the lysosome [96]. Here, it binds to the lysosomal receptor LAMP-2A and facilitates, together with its co-chaperones, in the unfolding process of the substrate in order to translocate it into the lysosome [97]. 

Microautophagy is a process in which cytoplasmic cargo is directly engulfed into the lysosome [98]. This process is triggered by the same stimuli compared to common autophagy, but in contrast it mainly facilitates the degradation of smaller substrates, including misfolded proteins. Similar to CMA, Hsc70 is involved in the protein cargo selection for degradation [99]. 

## 3. Therapeutic Approaches to Restore Protein Balance

The light sensing OS of PRs requires renewal every ten days due to its high photoactivity, especially of the cones during daytime vision. This demands a high turnover of biosynthetic compounds, including membranes and proteins). Therefore, a well-balanced proteostasis network is of particular importance for the PRs. For many IRDs it has been shown that this balance in the PRs is disrupted, leading to protein accumulation in the IS and eventually the death of photoreceptors. For this reason, several studies have focused on therapeutically targeting the three main branches of the PR proteostasis network to restore the protein balance. The different therapeutic approaches and their effects on PRs are discussed below and summarized in Table 1.

### 3.1. Therapeutic Strategies Involved in Protein Trafficking

#### 3.1.1. Targeting Protein Lipid Modifications

Agents targeting prenylation have been extensively studied in cancer research. The first agents tested were FTase inhibitors used to target the farnesylation of K-Ras, which was shown to induce several forms of cancer, including colorectal cancer and lung cancer [142]. Although some clinical improvements were seen for different forms of cancer, these improvements could not be related to inhibition of K-Ras. Subsequent experiments revealed that K-Ras was geranylgeranylated when farnesylation was blocked by FTase inhibitors [143]. Therefore, inhibitors of GGTase-I emerged as potential target for cancer therapy. However, a phase I clinical trial investigating a dual FTase and GGTase-I inhibitor showed dose-limiting side effects in patients with locally advanced pancreatic cancer, indicating that inhibition of prenylation has a broad detrimental effect on human health [102]. FTase inhibitors are also of particular interest in the Hutchinson–Gilford progeria syndrome (HGPS) research field. HGPS is a rare premature-aging disease caused by a dominant de novo point mutation in the *LMNA* gene, resulting in the expression of a mutant form of lamin A, also known as progerin [144]. Progerin remains persistently farnesylated, which makes it a suitable candidate for FTase inhibitors [100]. Although, promising improvements in bone structure, vascular stiffness, and hearing have been obtained using FTase inhibitors in HGPS patients, nothing is reported about possible dose-limiting side effects [101].

Despite the promising results of using FTase and GGTase-I inhibitors for targeting several forms of cancer and HGPS, the side effects are of major concern; especially in the highly compartmentalized PRs, where many proteins depend on prenylation for subcellular trafficking and anchoring. General inhibition of prenylation would probably lead to underprenylation of many PR proteins leading to mislocalization and accumulation of these proteins in the IS. Therefore, inhibition of prenylation in PRs will rather accelerate protein accumulation than decreasing it.

### 3.2. Therapeutic Strategies Involved in Protein Folding

#### 3.2.1. Targeting Chaperonins and Their Co-chaperones

The type II chaperonin CCT is proposed to be involved in the folding of approximately 10% of newly translated cytosolic proteins [145]. Besides ATP, CCT also needs the co-chaperone PhLP1 for the correct folding of these proteins [47]. It has been shown that inhibition of CCT by the transgenic expression of a dominant-negative mutant of PhLP1 results in the malformation of the OS in mouse photoreceptors [103]. Proteomic analysis revealed that the expression of several important PR proteins was affected, including PDE6, rhodopsin, transducin, peripherin 2, ROM1, musashi-1, and UNC-119. It could be possible that CCT is responsible for the folding of these proteins. Yet, only transducin has been shown to be a direct substrate of CCT. Therefore, a more plausible explanation would be that PhLP1 regulates the expression of these proteins in a CCT-independent manner.

Besides transducin, the BBSome is also a substrate for CCT for its correct folding and assembly. It has been shown that some of the BBSome proteins, including BBS6, BBS10, and BBS12, can form a CCT-like complex together with CCT subunits. Interestingly, 50% of clinically diagnosed BBS cases are caused by a mutation in one of these three genes. Furthermore, mutations in these genes result in a more severe phenotype compared to other BBS proteins [105].

These findings highlight the importance of chaperone defects as pathogenic factors and therefore as potential therapeutic targets. Surprisingly, chaperonins and their co-chaperones, such as PhLP1, have not been investigated in relation to therapy. Especially PhLP1 would be an interesting therapeutic target, because of its proposed CCT-dependent and CCT-independent regulation of important PR proteins [104]. 

In cancer research, RIC8, functioning as GEF and chaperone for G_α_, has been proposed as a potential therapeutic target, since it has been shown that constitutive activation of G_α_ by somatic mutations leads to development of various cancers [106]. Therefore, inhibition of RIC8 by small molecules might reduce tumorigenesis. A constitutive active mutant of rod G_α_ has also been associated to a form of congenital stationary night blindness [107]. RIC8 inhibition might therefore also alleviate or delay this phenotype. However, as described earlier, the function of RIC8 in the PR has not been investigated and inhibition of RIC8 would only be applicable to this specific mutation.

#### 3.2.2. Targeting the Heat Shock Response (HSR)

HSP90, a component involved in the HSR, is commonly overexpressed in multiple forms of cancer [146]. It has been proposed that cancer cells increase their HSP90 expression and thereby change their proteostasis network in order to adapt to dysregulated and misfolded protein synthesis as a consequence of rapid cell division [147]. For this reason, many studies have focused on investigating HSP90 inhibitors. The first HSP90 inhibitor identified was geldanamycin, but this never reached the clinical testing phase because of its poor applicability and toxicity [108]. Nevertheless, geldanamycin paved the way for production of geldanamycin analogs, including 17-AAG/tanespimycin and 17-DMAG/alvespimycin [109]. Although clinical testing was halted for these agents, it did provide proof-of-principle that HSP90 is a relevant target for cancer therapy. Now, a new generation of HSP90 inhibitors, including luminespib, onalespib, and ganetespib, show greater potency in clinical testing [109].

HSP90 inhibitors have also been tested in models for retinal degeneration. Systemic administration of 17-AAG accompanied by inner-blood retina barrier modulation in a mouse model of RP (RP10) protects against photoreceptor degeneration induced by aggregating RP10-associated mutant IMPDH1 protein [110]. Furthermore, HSP90 inhibition by 17-AAG in a transgenic rat model of RP (R135L) reduced the intracellular accumulation of the mutant protein and restored the localization of this protein comparable to wild type controls [111]. Administration of another HSP90 inhibitor, HSP990, in a different transgenic rat model of RP (P23H) resulted in enhanced visual function and delayed photoreceptor degeneration. However, prolonged treatment with HSP990 leads to a reduction in GRK1 and PDE6 protein levels followed by visual impairment [111]. The visual impairment caused by prolonged HSP90 inhibition is in line with findings from a study in which four HSP90 inhibitors were tested, including 17-AAG, 17-DMAG, luminespib, and ganetespib, in a rat retinal damage model. Here, it was shown that prolonged inhibition with 17-DMAG and ganetespib results in PR cell death, whereas there was no PR injury detected upon treatment with the other two agents [112].

These contradictory findings might be explained by the different modes of action of the HSP90 inhibitors, since the effect of 17-AAG was dependent on HSF1 in the P23H rat model, whereas its effect was HSF1-independent in the R135L model. This is further supported by the notion that HSP90 is known to facilitate the folding and assembly of more than 300 proteins [146]. Alternatively, agents that induce the HSR in other ways might be more suitable as potential treatments for IRDs. A direct way to target the HSR is by overexpressing the *HSF-1* gene. A study in the p23H RHO RD rat model showed a significant increase of scotopic electroretinogram (ERG) amplitudes compared to untreated controls upon ectopic overexpression of *HSF-1* by subretinal injection of AAV-*H*SF-*1* [113].

The hydroxylamine derivative arimoclomol might be a more indirect alternative agent to induce the HSR. Treatment of transgenic rats, carrying a P23H rhodopsin mutation, with arimoclomol resulted in improved PR OS structure and reduced rhodopsin accumulation in the IS, accompanied by prolonged PR survival and improved visual responses [114]. It was shown that these improvements were mediated by both the HSR and the UPR, thereby indicating that these responses are intertwined. Another great advantage of arimoclomol is that it only acts on stressed cells. In this way, potential side effects, as a consequence of inducing an HSR response in healthy cells present in the retina, are avoided.

#### 3.2.3. Targeting the Unfolded Protein Response (UPR)

As described earlier, the UPR senses ER stress by using three transmembrane sensors, including IRE1, PERK, and ATF6. In IRDs, mutations causing defective proteins can result in prolonged ER stress. It has been shown that upon prolonged ER stress, downstream effectors of the PERK pathway, ATF4 and CHOP heterodimerize. This results in increased protein synthesis, protein misfolding, and oxidative stress, eventually leading to cell death [148]. Another branch of the UPR that gets hyperactivated upon prolonged ER stress is IRE1. Hyperactive IRE1 cleaves microRNAs that normally inhibit proapoptotic targets, thereby inducing apoptosis [149]. Because apoptosis as a consequence of ER stress is often seen in PRs of IRD patients, therapeutically targeting UPR regulators or pathways involved in one of the three branches of the UPR might promote PR survival and thereby visual function.

As discussed earlier, it is commonly accepted that BiP/GRP78 is the key regulator in the activation of the UPR. Therefore, BiP might be a potential therapeutic target for relieving ER stress. Indeed, studies have shown that subretinal delivery of AAV5-BiP to a transgenic rat model of RP (P23H) results in reduction of PR cell death and improved ERG amplitudes [115]. Furthermore, a study in primary human retinal pigmented epithelium cells (hRPE) showed that AAV2-BiP promotes the survival of these cells under ER stress [116]. Both studies suggest that these improvements are due to a suppression of apoptosis by downregulation of CHOP protein levels. For this reason, reducing CHOP levels might be a promising strategy to alleviate ER stress. This notion is supported by a large body of evidence in the field of Alzheimer’s Disease, cardiac hypertrophy, and diabetes, wherein they link CHOP to these disease conditions [150].

Recently, several studies investigated the effect of CHOP reduction in the context of photoreceptor degeneration. A first study was performed in a transgenic mouse model of RP (T17M) [117]. Subsequently, these mice were crossed with CHOP knockout mice in order to get T17M RHO CHOPP^−/−^ mice. Complete knockdown of CHOP in these mice resulted in photoreceptor cell death, indicated by significant thinning of the outer nuclear layer, accompanied by a strong impairment in visual function. In a similar experimental setup comparing P23H RHO CHOPP^−/−^ mice with P23H RHO mice, they found no effect on PR survival in young animals. In older mice, however, the central retina of the CHOPP^−/−^ mice was partly protected against degeneration [118]. Another study in transgenic mice expressing human P23H rhodopsin also showed that knockout of CHOP had no effect on the rescue of retinal degeneration [119]. These studies indicated that complete ablation of CHOP has no positive effect in early developmental stages. As already described earlier, CHOP is mainly involved in prolonged PERK activation. Therefore, reducing CHOP levels to physiological levels during prolonged ER stress might be a better solution. This notion is supported by above described findings in older mice.

Interestingly, knocking down the proapoptotic partner of CHOP, ATF4 in a T17M rhodopsin mutant mouse model resulted in decreased retinal degeneration and improved PR survival [120].

Further support that the PERK branch of the UPR might be a promising therapeutic target comes from studies investigating ER stress related diseases, including Alzheimer’s Disease (AD), amyotrophic lateral disease (ALS), Parkinson’s Disease, and prion diseases [151,152].

In an Alzheimer’s disease mouse model it was shown that deletion of PERK resulted in decreased phosphorylation of eIF2α and prevented deficits in protein synthesis leading to an improvement of the AD phenotype in these mice [153]. Comparable results were obtained in prion-diseased mice by overexpressing GADD34, which plays a role in the negative feedback loop for PERK by acting as a cofactor in the dephosphorylation of eIF2α [154]. Treatment of the prion-diseased mice with salubrinal, an inhibitor of eIF2α dephosphorylation, had the opposite effect compared to GADD34 overexpression, thereby supporting the positive effects of eIF2α dephosphorylation. The positive effect of the inhibition of the PERK pathway is further supported by a study investigating the effect of the specific PERK inhibitor, GSK2606414A, in prion-diseased mice [155]. Treatment with this inhibitor showed reduced neuronal cell death leading to increased survival of these mice.

Surprisingly, opposing results were found for GSK2606414A and salubrinal treatment when administered in P23H RHO transgenic rats. GSK2606414A treatment resulted in accelerated photoreceptor cell death and further impaired visual function, whereas salubrinal treatment was found to improve photoreceptor survival [121]. It could be possible that in some disease conditions or models the PERK pathway is protective, whereas in other conditions it is rather a secondary effect of the disease process. Therefore, targeting other branches of the UPR might overcome this issue.

Targeting the IRE1 branch of the UPR has been shown to be beneficial in multiple rodent models, including a P23H RHO rat model [122]. In this model, intravitreal injection of KIRA6, which allosterically inhibits the RNase domain of IRE1, showed increased photoreceptor survival.

Recently, an emerging role of reactive electrophilic species (RES) as key regulators for the UPR, more specifically the IRE1 pathway, have been described [123]. Furthermore, RES have been reported to be involved in ER-stress related diseases, including neurodegenerative diseases, amyotrophic lateral sclerosis (ALS), and cancer [124,125,126]. The RES nitric oxide (NO) has been shown to modulate PDI and IRE1 through S-nitrosylation and thereby inhibiting the IRE1 branch of the UPR [127,128]. Therefore, modulation by RES might be a new potential therapeutic strategy for ER stress-associated diseases, including IRDs.

Maybe the most interesting sensor of the UPR, in context of IRDs, is ATF6, because mutations in ATF6 have been shown to cause autosomal recessive achromatopsia and early onset photoreceptor degeneration, also affecting the macula [156,157].

Until recently, no compounds were available that specifically target the ATF6 branch of the UPR. However, by cell-based screens a new class of pyrazole amides, named Ceapins, were identified as selective inhibitors of ATF6α [129,130]. Ceapins selectively prevent transport of AFT6α to the Golgi apparatus during ER stress. Interestingly, Ceapins have no toxic impact on unstressed cells, whereas they increase ER stress sensitivity upon ER stress induction.

### 3.3. Therapeutic Strategies Involved in Protein Degradation

#### 3.3.1. Targeting the ER-associated Degradation (ERAD)

One of the essential players in ERAD is p97/VCP, belonging to the type II AAA+ protein family of ATPases. Because of its essential role in ERAD, VCP is proposed as a novel therapeutic target for the treatment of various diseases, including cystic fibrosis, cancer, and neurodegenerative disorders [131]. Besides its function in retrotranslocation and protein degradation, VCP is also involved in many other cellular processes, including mitosis, nuclear reformation, DNA/RNA repair, aggresome formation and inflammatory signaling [158]. Therefore, for therapeutic applicability, selective intervention of VCP is required to minimize the side effects. Small compounds, named Kyoto University Substances (KUSs), might fulfill these criteria. KUSs have been shown to specifically inhibit VCP’s ATPase activity, without affecting the cellular functions of VCP [131]. Administering KUSs, more specifically KUS121 or KUS187, by intraperitoneal injection in a *rd10* mouse model resulted in preserved outer nuclear layer (ONL) thickness and improved visual function measured by ERG.

Because of the unfolding properties of VCP also other AAA+ proteins have been proposed as potent therapeutic agents, especially in solving protein misfolding and aggregation in neurodegenerative diseases. Although not functionally related to ERAD, the repurposing of two AAA+ proteins, including Hsp104 and proteasome-activating nucleotidase (PAN) were shown to protect from protein misfolding in several neurodegenerative animal models [159]. Furthermore, transgenic expression of PAN in rods of mice lacking the γ-subunit of transducin (G_γ_^−/−^) resulted in increased photoreceptor survival accompanied by preserved visual function [132]. 

Another possible therapeutic target of ERAD could be the enhancement of proteosomal degradation. Important players in this process are the deubiquitination enzymes (DUBs), also known as ubiquitin-specific proteases (USPs). USPs are known to cleave ubiquitin moieties from proteins and thereby preventing these proteins from proteosomal degradation [133]. Therefore, inhibiting USPs might work as a therapeutic approach to stimulate the degradation of misfolded proteins by the proteasome. In cancer research inhibition of specific USPs, including USP7 and USP10, has already been shown to have beneficial effects, by acting on the tumor suppressor p53 [134].

Depending on the type of mutation, p53 can either gain oncogenic properties or lose its tumor suppressor ability [160]. Therefore, based on the mutant form of p53, either increasing or lowering the levels of p53 could be beneficial. Inhibiting USP7, a negative regulator of p53, leads to increased p53 levels, whereas inhibiting USP10, a positive regulator of p53, leads to reduction of p53 levels [134]. With this in mind, the causative mutation for the disease has to be known, as well as the specificity of the USP inhibitor before administering it to the patient. From discrepancies in findings of different studies it cannot be concluded whether p53 is involved in the pathogenesis of PR cell death [161,162]. However, a recent study found a biallelic mutation in *USP45* associated with LCA. This indicates that USPs can play important roles in the pathogenesis of PR degeneration [163].

Another important issue to address is that DUBs associated with VCP have been suggested to promote protein turnover by assisting in retrotranslocation instead of their traditional function in preventing proteasomal degradation. Therefore, inducing these specific VCP associated DUBs might have therapeutic potential [135].

#### 3.3.2. Targeting Autophagy

One of the best known inducers of autophagy is rapamycin, which directly binds to mTORC1, thereby inhibiting the mTOR pathway [164]. Because of its limited absorption, studies have been focusing on investigating rapamycin analogs or rapalogs, including everolimus, temsirolimus, and ridaforolimus. These mTORC1 inhibitors were highlighted as promising therapeutic agents in various protein misfolding associated diseases, including neurodegenerative diseases and multiple forms of cancer [165,166]. Also, a study in an RP rat model has shown the therapeutic potential of rapamycin in treating blindness [136]. In this study, rod cell degeneration was slowed down, without affecting cones, in a P23H rat model upon systemic administration of rapamycin. In contrast, other studies showed that activation of the mTOR pathway promotes cone survival [167,168,169]. Taken together, these results indicate that activation of autophagy is particularly protective for rods, but can have opposing effects on cones. 

Studies investigating other mTOR inhibitors, including ATP-competitive mTOR inhibitors, are emerging [170]. Furthermore, indirect modulators of the mTOR pathway, including metformin and nilotinib have also been shown to play a protective role in diabetes, cancer, and neurodegenerative diseases via indirect activation of AMPK [171,172,173]. Contradictory results are found in mouse models of retinal degeneration when treated with metformin. In diabetic mice, metformin treatment protected against retinal cell death, whereas in a P23H RHO mouse model it accelerated the photoreceptor degeneration, indicating different modes of action by metformin [137,138]. 

In addition to autophagy upregulators acting on the mTOR pathway, also mTOR-independent autophagy upregulators have been widely studied, mainly in Huntington’s disease [174]. One such agent is valproic acid (VPA), which upregulates autophagy by inhibiting inositol synthesis. In line with findings in neurodegeneration models, VPA treatment in models for photoreceptor degeneration can be detrimental or protective [139,175]. In a mouse model for Bardet–Biedl syndrome (BBS12^−/−^), as well as in a *Xenopus laevis* model for RP (P23H) treatment with VPA resulted in photoreceptor protection [139,140]. In contrast, a T17M *X. laevis* model of RP and the P23H-1 rat model showed exacerbated photoreceptor degeneration upon VPA treatment [15,139]. Even in mouse models with a different mutation in the same gene (PDE6) this discrepancy in outcome was observed: VPA treatment reduced photoreceptor loss in the *rd1* model, whereas VPA treatment slightly accelerated photoreceptor loss in the *rd10* model [141]. Taken together, these studies indicate that the outcome of VPA treatment depends on the genotype and even on the type of mutation. The conflicting results might be partly explained by the different modes of action by VPA, since VPA is also acting as a histone deacetylase (HDAC) inhibitor, thereby altering expression of different genes [176].

## 4. Discussion and Outlook

The OS of the PR can be considered as a highly specialized cilium responsible for the conversion of light stimuli into electrical signals, also known as the phototransduction cascade. This cascade requires a tremendous amount of energy as well as a continuous OS protein turnover to maintain cellular homeostasis. Therefore, a perfectly balanced proteostasis network is of particular importance for the PRs. In many IRDs this balance is disrupted leading to protein accumulation in the IS and ultimately to PR cell death. Several attempts were made to restore this balance by therapeutically targeting different branches of the proteostasis network, including protein trafficking, folding, and degradation. For therapeutic intervention, it has to be taken into account that these different branches consist of tightly intertwined processes, which depend on each other and influence each other’s function and activation. For example, administration of the AMPK activator metformin to P23H RHO mice resulted in enhanced trafficking of rhodopsin to the rod OSs. However, it still led to reduced PR function and survival. Apparently, metformin did not succeed in full restoration of the correct folding of rhodopsin, leading to increased destabilization of the OSs [137]. Taken together, enhancing protein trafficking without improving protein folding will accelerate the disease progression rather than diminishing it. Recently, also an intimate cross-talk between the UPR, more specifically IRE1, and ERAD has been described [177]. On one hand, splicing of XBP1s by activated IRE1 promotes the expression of ERAD components, while on the other hand ERAD serves as a feedback loop for IRE1 by targeting it for degradation. This cross-talk is probably further extended to other branches of the UPR, since it has been shown that the turnover of ATF6 is also regulated by ERAD [178]. 

Another issue that has to be taken into account for therapeutic intervention is that in some disease conditions certain branches of the proteostasis network are protective, whereas in other disease conditions it is a consequence of the disease process. Therefore, it is often not clear which branch of the proteostasis network would be the most effective target to treat a certain disease type. Even different mutations in the same gene causing similar disease conditions can have an effect on the treatment outcome, indicated by contradictory findings in VPA treatment in the *rd1* and *rd10* mouse models [141]. This discrepancy might be partly explained by the severity of the gene mutations leading to different rates of disease progression, since *rd1* mice show rapid degeneration of PRs, whereas the *rd10* mouse model represents a more slowly progressive form of RP. With this in mind, therapeutic compounds that have an positive effect on slow progressing forms of RP might not be suitable for fast progressing forms of RP and early onset forms of blindness, such as LCA.

The animal model used for therapeutic testing, especially animals carrying a transgene, can also influence therapeutic outcome. One such animal model carrying a transgene is the P23H RHO rat model. Numerous different lines with various transgene expression exist [179,180,181]. It has been shown that the amount of expression of the transgene can have an effect on the disease phenotype. Even overexpression of wild type human rod opsin has been shown to induce PR degeneration [182]. In contrast, it has been shown that overexpression of wild type RHO partially rescues visual responses in a P23H RHO rat model [113]. Nevertheless, studies using the P23H RHO rat model presented in this review should be interpreted with caution, especially when translating therapeutic outcome to the patient situation. 

Instead of directly targeting the different branches of the proteostasis network, mimicking the actions of these branches might also be a valuable therapeutic approach. The main action of the PERK pathway of the UPR is reducing the protein synthesis and thereby reducing the ER-stress load. This action can also be regulated by targeting the epigenome, including histone acetylation. Enzymes that play an important role in this acetylation process are the histone deacetylases (HDACs). Inhibiting these HDACs was shown to be protective for PR cell death. Administration of the HDAC6 inhibitor tubastatin A in an inherited blindness zebrafish model resulted in the rescue of retinal morphology and visual function [183]. However, further investigation of the underlying molecular mechanisms is necessary, since poor efficacy and safety have been reported for other HDAC inhibitors, including VPA [176,184].

Another way to influence protein synthesis is by the regulation of splicing. The importance of splicing in the retina is indicated by mutations found in the ubiquitously expressed pre-mRNA processing factors (PRPFs), which only cause retinal-specific degeneration [185]. In situ gene editing of a pathogenic, dominant mutation in PRPF31 resulted in the rescue of protein expression as well as the phenotype [186]. The proof-of-principle for targeting splicing as potential therapeutic strategy is further supported by the application of antisense oligonucleotides for the treatment of inherited retinal diseases, including CEP290 associated LCA [20,187]. 

In addition to influencing protein synthesis, degeneration of PRs, which is the ultimate consequence of sustained ER-stress and a key hallmark of IRDs, can also been seen as a therapeutic target. A broad range of therapeutic agents is available that can be protective for PR cell death, including cell death inhibitors, caspase modulators, and neurotrophic factors. Individual treatment of P23H-1 rats with the known cell death inhibitors calpeptin, N-acetylcysteine, and necrostatin-1s all resulted in improved photoreceptor function [188]. Furthermore, the chemical chaperone tauroursodeoxycholic acid (TUDCA), which inhibits apoptosis by preventing BAX from being transported to the mitochondria to initiate caspase release, has been shown to preserve cones in a LCA mouse model upon systemic injection [189]. On top of that, direct inhibition of caspases by subretinal AAV delivered X-linked inhibitor of apoptosis (XIAP) in two RP rat models protected PR structure and function [190]. Finally, neurotrophic factors, including CNTF, BDNF, GDNF, LEDGF, PEDF, and RdCVF were also found to have positive effects on PR survival [191]. However, the therapeutic potential of neurotrophic factors as a general treatment may not outweigh the plausible risk of side effects. These side effects can be reduced by carefully selecting the proper administration route, which accounts for every therapeutic agent described in this review. In general, there are three possible delivery methods to reach the eye: systemic, topical, and local. Each of these methods have their advantages and disadvantages [192]. Systemic delivery is often accompanied by systemic side effects and drugs need to pass the blood–retinal barrier first to perform their function, but it is less invasive compared to intraocular injections. Topical delivery in form of eye drops or ointments is not invasive, but the chance that the therapeutic compound reaches its destination is often an issue. Intraocular injection is the most invasive delivery method, but most often also the most effective one. Furthermore, with this method the involvement of the immune system is largely circumvented, as the eye is a largely immune-privileged organ, thereby further reducing the risk of potential side effects. Intraocular delivery can be performed by injecting the therapeutic compound by itself, but the cell penetrance and thus delivery efficiency is much higher when the compound is packed into nanoparticles, for example [192]. 

Immense progress has been made in optimizing the delivery methods in combination with the drug formulation, especially for gene augmentation and antisense-oligo nucleotide (AON)-based treatments. Now, an increasing number of studies are aimed at developing broader applicable treatments, and therefore are focused on therapies targetting the proteostatis network. Many of these studies show hopeful results in delaying blinding conditions [111,114,121,122,131]. However, much effort has still to be made to first efficiently halt the progression of IRD, let alone fully restore vision.

## Figures and Tables

**Table 1:** Therapeutic compounds directed against the different branches of the proteostasis network (PN). Therapeutic compounds are divided based on which branch of the PN they target, including trafficking (green), folding (blue), and degradation (red). Subgroups include compounds directed against protein lipid-modifications, (co-)chaperonins, HSR, UPR, ERAD, and autophagy. * Compounds that are not yet available.

Target	Compounds *	Function	Effect on PRs	References
Protein lipid-modifications	FTase inhibitors	Inhibits the farnesylation of proteins	(Proposed) underprenylation and mislocalization of many PR proteins	[100,101]
	GGTase-I inhibitors	Inhibits the geranylgeranylation of proteins	(Proposed) underprenylation and mislocalization of many PR proteins	[102]
(Co-)chaperonins	CCT inducers *	Improves folding of transducin and possibly other PR proteins	Undetermined	[103]
	PhLP1 inducers *	Improves function of CCTs and possibly CCT-independent functions	Malformation of OS by transgenic expression of a PhLP1 dominant-negative mutant	[47,103,104]
	CCT-BBSome stabilizers *	Stabilizes BBSome formation and thereby the export of molecules from the OS	Undetermined	[105]
	Small molecule Ric8 inhibitors *	Prevents folding of disease-causing Gα	Undetermined	[106,107]
Heat shock response (HSR)	geldanamycin, tanespimycin, alvespimycin	1st generation HSP90 inhibitors	Geldanamycin not suitable for future experiments because of poor applicability and toxicity; Tanespimycin reduced mutant protein accumulation in rat RP model (R135L); prolonged treatment with alvespimycin leads to PR cell death	[108,109,110,111,112]
	luminespib, onalespib, ganetespib, HSP990	Newer generation HSP90 inhibitors	HSP990 treatment in a RP rat model (P23H) enhances visual function and delayed PR degeneration, but prolonged treatment led to visual impairment by GRK1 and PDE6 reduction; prolonged treatment with ganetespib led to PR cell death	[109,111,112]
	AAV-*HSF-1*	Overexpressing HSF-1 and thereby transcriptional activation of HSPs	Subretinal injection of AAV-*Hsf-1* in a RP rat model (P23H) improved visual reponse	[113]
	Arimoclomol	Induces HSR and UPR, only in stressed cells	Prolonged PR survival and improved visual responses in P23H transgenic rats	[114]
Unfolded protein response (UPR)	AAV-BiP	Relieves ER stress by reducing cleaved ATF6, phosphorylated eIF2α and CHOP	Subretinal delivery of AAV5-BiP reduced PR cell death and improved visual responses in P23H transgenic rats	[115,116]
	CHOP inhibitors *	Inhibits proapoptotic transcription activity of CHOP	CHOP knockout in a trangenic mouse model of RP (T17M) led to PR cell death and strong impairment in visual function; CHOP knockout in P23H RHO mice had no effect on PR survival in young mice, but partly protected PR degeneration in older mice	[117,118,119]
	ATF4 inhibitors *	Inhibits downstream transcription activation of Chop, Ero1, and Gadd34	ATF4 knockdown in T17M RHO mice decreased retinal degeneration and improved PR survival	[120]
	GSK2606414A	Specific PERK inhibitor	Treatment with GSK2606414A in P23H RHO rats accelerated PR cell death and further impaired visual function	[121]
	Salubrinal	Inhibitor of eIF2α dephosphorylation	Treatment with salubrinal in P23H RHO rats improved PR survival	[121]
	KIRA6	Allosterically inhibits IRE1α RNase activity	Intravitreal injection of KIRA6 in P23H RHO rats increased PR survival	[122]
	Reactive electrophilic species (RES) modulators *	Modulate the effects of RES on IRE1	Undetermined	[123,124,125,126,127,128]
	Ceapins	Selective inhibitors of ATF6α	Undetermined	[129,130]
ER-associated degradation (ERAD)	Kyoto University Substances (KUSs)	Inhibits VCP’s ATPase activity, without affecting the cellular functions of VCP	Individual treatment with KUS121 and KUS187 in a *rd10* mouse model preserved ONL thickness and improved visual function	[131]
	AAA+ protein derivatives *	Unfolding of misfolded proteins	Transgenic expression of PAN in Gy-/- mice increased PR survival and preserved visual function	[132]
	DUB/USP modulators	Modulating the ubiquitin cleavage from proteins, thereby modulating proteosomal degradation	Undetermined	[133,134,135]
Autophagy	Rapamycin, everolimus, temsirolimus, ridaforolimus	Inhibiting mTOR pathway by directly binding to mTORC1	Improved rod survival in P23H-3 rats	[136]
	Metformin	Activation of AMP-activated protein kinase (AMPK)	Metformin treatment protected against retinal cell death in diabetic mice, whereas it accelerated PR degeneration in P23H RHO mice	[137,138]
	Valproic acid (VPA)	Upregulates autophagy by inhibiting inositol synthesis	VPA treatment in BBS12-/- mice, P23H RHO *Xenopus laevis*, and a *rd1* mouse model resulted in PR protection, whereas treatment in T17M RHO *X. laevis*, a P23H-1 rat model, and a *rd10* mouse model excerbated PR degeneration	[139,140,141]
